# Broadband activation by white-opsin lowers intensity threshold for cellular stimulation

**DOI:** 10.1038/srep17857

**Published:** 2015-12-14

**Authors:** Subrata Batabyal, Gregory Cervenka, David Birch, Young-tae Kim, Samarendra Mohanty

**Affiliations:** 1 Biophysics and Physiology Lab, The University of Texas at Arlington, TX, USA; 2 Retina Foundation of the Southwest, Dallas, TX, USA; 3 Department of Bioengineering, The University of Texas at Arlington, TX, USA

## Abstract

Photoreceptors, which initiate the conversion of ambient light to action potentials via retinal circuitry, degenerate in retinal diseases such as retinitis pigmentosa and age related macular degeneration leading to loss of vision. Current prosthetic devices using arrays consisting of electrodes or LEDs (for optogenetic activation of conventional narrow-band opsins) have limited spatial resolution and can cause damage to retinal circuits by mechanical or photochemical (by absorption of intense narrow band light) means. Here, we describe a broad-band light activatable white-opsin for generating significant photocurrent at white light intensity levels close to ambient daylight conditions. White-opsin produced an order of magnitude higher photocurrent in response to white light as compared to narrow-band opsin channelrhodopsin-2, while maintaining the ms-channel kinetics. High fidelity of peak-photocurrent (both amplitude and latency) of white-opsin in response to repetitive white light stimulation of varying pulse width was observed. The significantly lower intensity stimulation required for activating white-opsin sensitized cells may facilitate ambient white light-based restoration of vision for patients with widespread photoreceptor degeneration.

Degeneration of retinal photoreceptors eliminates the light-stimulated neuronal activity necessary to drive retinal circuitry, activate cortical circuits, and mediate visually guided behaviors^1^,^2^,^3^,^4^. Progressive photodegenerative disorders are the leading cause of new vision loss in millions of individuals^5^,^6^,^7^. Most of the current clinical treatments are primarily focused on slowing down the progression of the disease^4^,^6^,^8^. Partial restoration of vision has been successful, but involves an invasive surgical procedure to implant retinal electrodes^9^,^10^,^11^,^12^. The disadvantages of retinal implants include chronic damage from the implanted electrodes, insufficient current produced by microphotodiode (in case of subretinal implant) from the ambient light to stimulate adjacent neurons^13^,^14^, cellular outgrowth due to surgical implantation, and disordered stimulation patterns resulting from the electrical stimulation of both the axon and soma (in epiretinal implant)^14^. Further, electrical stimulation-based implants are limited by poor resolution (higher electrode densities require more current, leading to heat production) and do not provide cell-specific activation.

Optogenetic sensitization (e.g. channelrhodopsin-2, ChR2; halorhodopsin, NpHR) of higher-order retinal neurons or residual receptors, and cell-specific activation/inhibition with high temporal precision^15^,^16^,^17^,^18^,^19^ has potential as an alternative to electrical stimulation through implants. Optogenetics has advantages such as cellular specificity (e.g. residual cones, ganglion or bipolar cells), minimal invasiveness^20^,^21^ and elimination of intraocular surgery. Optogenetic activation of ChR2 has been evaluated for vision restoration in mice models of retinal degeneration either by non-specific stimulation of retina^22^ or in a cell-specific manner for retinal ganglion cells^23^,^24^,^25^,^26^,^27^ and ON bipolar cells^28^,^29^. Further, use of such active light stimulation of chloride-channel opsin (NpHR) expressing in longer-persisting cone photoreceptor protein has shown promise for restoration of vision^30^. However, clinical translation of optogenetic activation for vision restoration has a major challenge: due to narrow spectral sensitivity of conventional opsins, the light levels needed to activate the cells are at least an order of magnitude higher than bright ambient white light levels (~0.01 mW/mm^2^). Thus, intense narrow-band light sources^31^ are being developed to activate opsin-sensitized cells in retina for restoring vision. However, chronic stimulation of narrow-band opsin-expressing retinal cells at high light intensity may substantially damage the residual light-sensing function that might exist in the partially-degenerated retina.

Herein, we introduce an ambient white-light based cell stimulation paradigm employing a broad-band (400–700 nm) activatable white-opsin. Our results show that significant photocurrent with ms-channel kinetics can be reliably generated in white-opsin sensitized cells at white light intensity level close to ambient day-light condition. The significantly reduced white light stimulation intensity required for activating white-opsin sensitized cells will lead to ambient white light based restoration of vision in case of retinal degenerative diseases. This will provide high resolution vision restoration and eliminate the requirement of active-stimulation device.

## Results

### Principle of enhancing sensitivity for white-light activation using white-opsin

For enhancing sensitivity of cells towards ambient white light, a fusion vector containing three plasmids (connected by linker sequences) encoding opsins with spectrally-separated activation peaks (ChR2 in blue, C1V1 in green and ReaChR in red, schematic of activation spectra drawn based on published literature^32^,^33^, Suppl. Fig. 1a) was constructed, named white-opsin. The white-opsin is a fused protein chimera^34^, wherein the C-terminus of ChR2-YFP is connected via attBr linker to the N-terminus of C1V1. Similarly C1V1 and ReaChR are connected. Cells expressing broadband excitable white-opsin would have significant enhanced sensitivity towards ambient white light as compared to cells expressing only one narrow-band opsin. Although one may argue that three different narrow-band opsin-encoding plasmids can be separately delivered into targeted cell(s), the same stoichiometric expression of the three spectrally-separated opsins in each cell cannot be guaranteed. The white-opsin was constructed using MultiSite Gateway® Technology^35^, which uses site-specific recombinational cloning to allow simultaneous cloning of multiple genes in a defined order. Suppl. Table 1 (and Suppl. Methods) summarizes the flanking att sites for the genes and primer sequences designed to create the attB sites for each gene used to construct the white-opsin (three connected opsins). Entry clone for each vector (ChR2, C1V1, ReaChR) to create attB sites for white-opsin expression vector is shown in Suppl. Fig. 1b. Suppl. Fig. 1c shows ligation map containing ChR2, C1V1, ReaChR, and CMV promoter sequence, realized via LR clonase reaction. The gel electrophoresis of white-opsin construct (10.6 kb) is conducted to confirm the restriction bands (8.0 kb and 2.6 kb) obtained via digestion using restriction enzyme Bgl II (Suppl. Fig. 1d).

### Membrane trafficking of white-opsin

**Figure 1:**
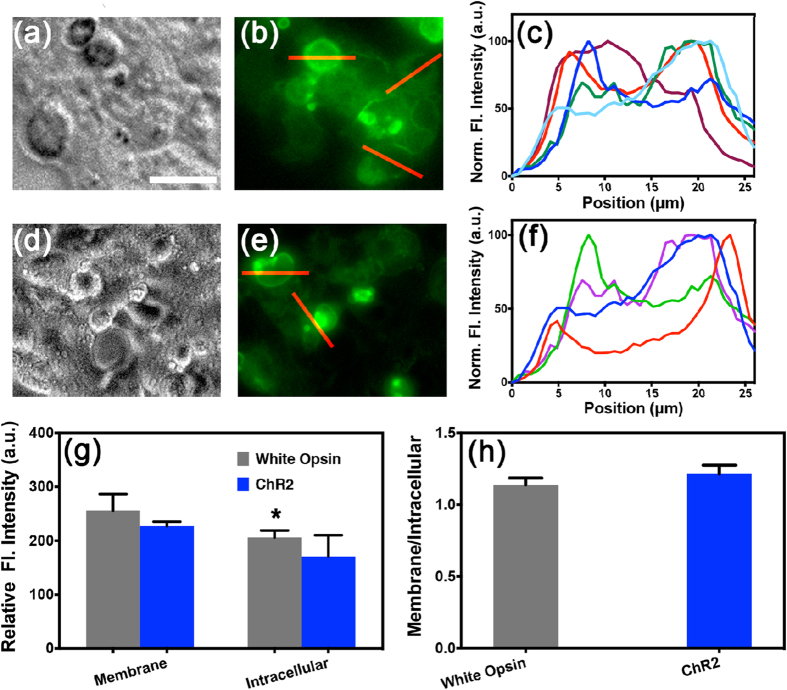
Expression of white-opsin is localized in plasma membrane. (**a**,**b**) Representative bright field and fluorescence images of HEK293 cells transfected with white-opsin-YFP. Scale bar: 15 μm. (**c**) White-opsin expression along line across representative cells (shown in **b**, red lines). (**d**,**e**) Representative bright field and fluorescence images of HEK293 cells transfected with ChR2-opsin-YFP. (**f**) ChR2-opsion expression along line across representative cells (shown in e, red lines). (**g**) Quantitative comparison of opsin expression on membrane and intracellular component between White-opsin (N = 6) and ChR2-opsin (N = 4). *p < 0.05 between membrane and intracellular component of white-opsin expressing cells. Average ± S. D. (**h**) Comparison of opsin-expression ratio of membrane to intracellular component: White-opsin versus ChR2 opsin. Average ± S.D. No statistical significant differences were found.

To enhance light sensitivity, ChR2-mutants are being developed and tested in HEK293 cells for evaluating their efficacy in vision restoration^36^,^37^. To evaluate membrane trafficking of white-opsin, we quantified the expression of white-opsin in cell membrane (vs. cytoplasm) of transfected HEK293 cells using fluorescence intensity of reporter protein (YFP and Citrine). The bright field and fluorescence images of HEK293 cells expressing white-opsin are shown in Fig. 1a,b respectively. No significant intracellular aggregation was observed implying effective trafficking of white-opsin to the plasma membrane. Further, to quantify the relative expression of the white-opsin in cell membrane and intracellular components, intensity profiles (along lines across cells, as shown in Fig. 1b) are plotted (Fig. 1c). The white-opsin expression in plasma membrane was significantly higher than intracellular expression. For determining whether the enhancement of photocurrent in white-opsin sensitized cells was due to its bandwidth sensitivity and not due to over-expression, we also quantified the expression levels for ChR2 in HEK293 cells (Fig. 1d,e). The ChR2-opsin expression along line across representative cells (shown in e, red lines) is shown in Fig. 1f. In Fig. 1g, we show the quantitative comparison of opsin expression in membrane and intracellular components between white-opsin and ChR2-opsin. No statistical significant differences were observed between ChR2 and white-opsin expression either in cell membrane or intracellular components. Further, the opsin-expression ratio (membrane to intracellular component) for white-opsin and ChR2 opsin was not statistically different (Fig. 1h). However, we have 3 reporter proteins (2 YFPs and 1 Citrine) per white-opsin (3 fused-opsins). Therefore, for same reporter-fluorescence intensity in ChR2 vs. white-opsin expressing cells, we have fewer white-opsin molecules in a white-opsin transfected cell than ChR2 molecules in ChR2-transfected cells.

### White-light intensity dependent peak current in white-opsin expressing cells

**Figure 2:**
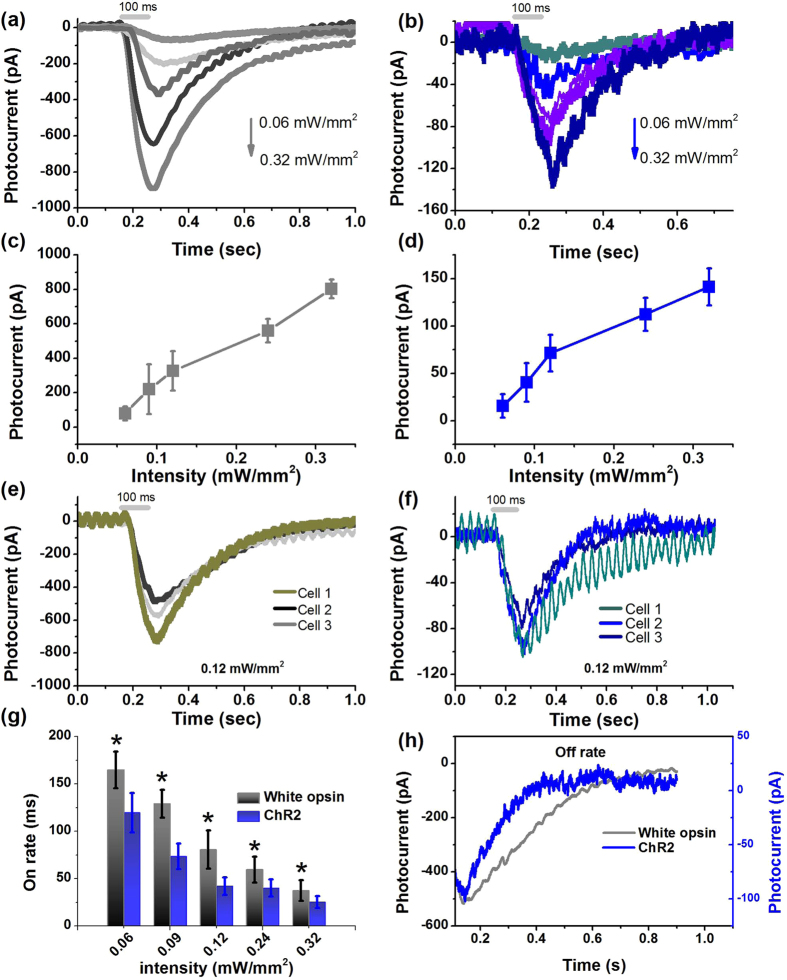
White-opsin expression leads to significantly higher photocurrent in response to white-light as compared to narrow-band ChR2-opsin. (**a**) Representative inward current in a white-opsin expressing HEK293 cell upon white light illumination at five different intensities. (**b**) Representative inward current in a ChR2 expressing HEK293 cell upon white light illumination at the same five different intensities. (**c**) Variation of peak photocurrent in white-opsin expressing HEK293 cells as a function of intensity of white-light (pulse width: 100 ms). N = 8 cells, 26 sweeps. Average ± S.D. (**d**) Variation of peak photocurrent in ChR2 expressing HEK cells as a function of white-light intensity. N = 8 cells, 27 sweeps. Average ± S.D. (**e**) White-opsin expression-dependent inward photocurrent measured in three different representative cells for fixed white light intensity. (**f**) Measured inward photocurrent in three different representative cells expressing different levels of ChR2 for same fixed white light intensity. (**g**) Quantitative comparison of on-rate of white-opsin and ChR2 in response to white light at five different intensities. N = 6 cells/opsins. *p < 0.01 between white-opsin and ChR2 at five different intensities. (**h**) Comparison of representative inward photocurrent decay (for off-response) measured in white-opsin and ChR2 expressing cell stimulated by same white-light intensity.

To determine the white-light intensity dependent peak current, the white-opsin-expressing cells were exposed to pulses (100 ms) of white light with intensity ranging from 0.06 to 0.32 mW/mm^2^ and the variation of the inward photocurrents were measured (Fig. 2a) using patch clamp electrophysiology (Suppl. Fig. 2). Suppl. Fig. 3 shows the measured spectrum of broadband white light used for optogenetic stimulation. With decrease in white light intensity, the inward current decreased linearly from ~800 pA to ~80 pA (Fig. 2a). The broad excitability of white opsin provides excitation by ambient white light at orders of magnitude lower spectral intensity (0.0002 mW. mm^−2^ nm^−1^) in contrast to narrowband active illumination, which requires a high spectral intensity density (~0.5–1.0 mW. mm^−2^ nm^−1^) for narrowband opsin (e.g. ChR2) activation^16^.

### White-opsin is more efficiently excited by white-light than ChR2

For evaluating the enhanced light-sensitivity of white-opsin as compared to narrow-band opsins, we transfected HEK293 cells with ChR2-YFP plasmids and subsequent electrophysiology experiments were carried out on the transfected cells. The opsin-expressing cells were exposed to pulses (100 ms) of white light with incident intensity ranging from 0.06 to 0.32 mW/mm^2^ and the inward current responses were measured by patch clamp (Fig. 2b). The peak photocurrent generated in ChR2-cells at white light intensity of 0.32 mW/mm^2^ was ~140 pA as compared to ~800 pA in white-opsin expressing cells. Similar to white-opsin, the peak photocurrent in ChR2 expressing HEK cells decreased linearly with reduced white-light intensity (Fig. 2c,d).

### Expression-dependent white-opsin photocurrent

For determining if white-opsin photocurrent is dependent on levels of expression, we correlated the (reporter-protein) expression levels in HEK293 cells with inward photocurrent induced by white light (Suppl. Fig. 4). The expression level was found to be higher for cells incubated longer after transfection. Three sets of bright and fluorescence images of white-opsin-YFP transfected HEK293 cells having three different expression levels after 24 hrs (top), 36 hrs (middle) and 48 hrs (bottom) of transfection is shown in left panel of Suppl. Fig. 4. In Fig. 2e, we show the representative inward photocurrent profiles upon white-light stimulation (100 ms, 0.12 mW/mm^2^) of cells at three different expression levels. Similar to white-opsin, expression levels dictated the inward photocurrent in ChR2-YFP transfected HEK293 cells. The measured inward photocurrent in three different representative cells expressing different levels of ChR2 (Right, Suppl. Fig. 4) for fixed white light intensity (100 ms, 0.12 mW/mm^2^) is shown in Fig. 2f.

### Intensity-dependent channel kinetics for white-opsin activation with white-light

Quantitative comparison of on-rate of white-opsin and ChR2 in response to white light at three different intensities (pulse width: 100 ms) was carried out. For both white-opsin and ChR2, the on-rate decreased with increase in white light intensity in the range studied here (0.06–0.32 mW/mm^2^). Though on-rate for white-opsin is higher than on-rate of ChR2, the fast ms kinetics is not severely compromised (Fig. 2g). The off-response of white-opsin was also found to be slower than ChR2 (Fig. 2h). With increase in white light intensity, the off-rate decreased.

### Effect of pulse-width of white-light stimulation on peak current and channel kinetics

**Figure 3:**
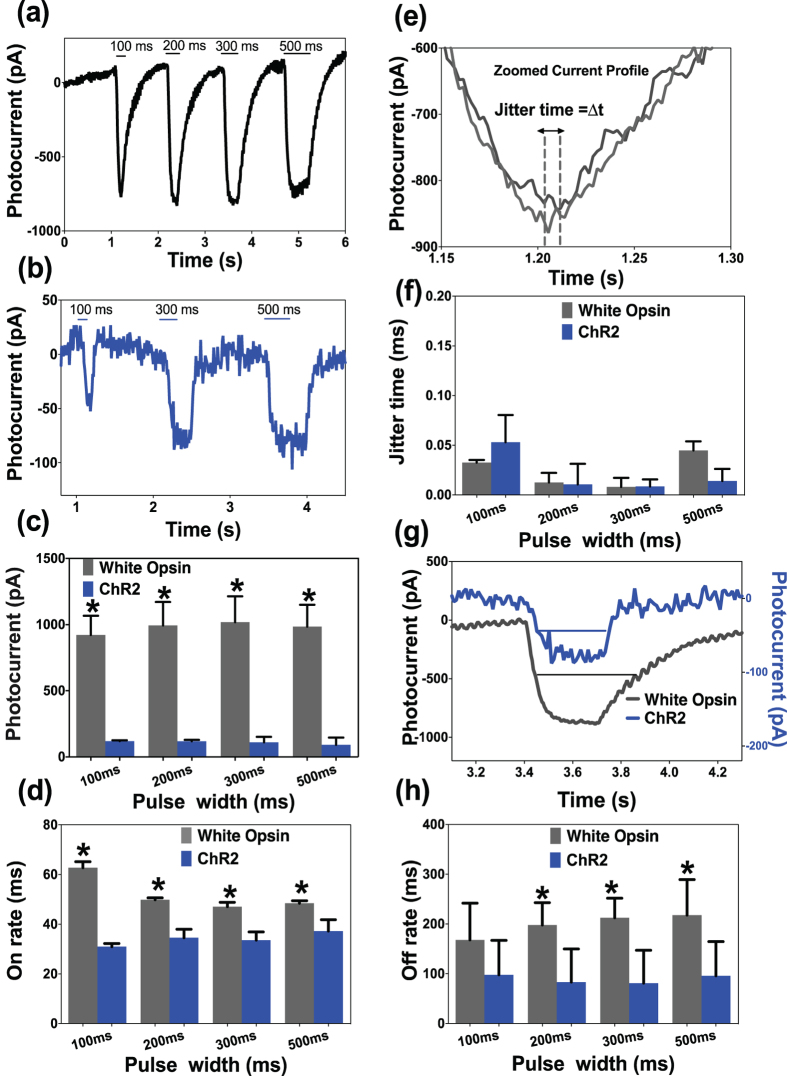
Dependence of peak current and on/off-rate on pulse-width of white-light stimulation of white-opsin expressing cells. Representative inward current in white-opsin (**a**) and ChR2 (**b**) expressing HEK293 cells upon white-light illumination with different pulse widths at fixed intensity. (**c**) Measured inward peak-photocurrent in white-opsin or ChR2 expressing cells as a function of pulse width of white-light. N = 5/opsins, 15 sweeps. *p < 0.01 between white-opsin and ChR2. No statistically significant difference between different pulse widths. (**d**) Quantitative comparison of on-rate of white-opsin and ChR2 in response to white light at four different pulse widths at fixed intensity. N = 5/opsins, 15 sweeps. *p < 0.01 between white-opsin and ChR2. (**e**) Method for measuring jitter in inward photocurrent in same cell at fixed stimulation parameters. (**f**) Quantitative comparison of jitter time for white-opsin and ChR2 at different pulse width of white-light. (**g**) Zoomed photocurrent for white-opsin and ChR2 showing different off-rates. (**h**) Off-rate for white-opsin and ChR2 as a function of pulse width of white-light. N = 7/opsins, 21 sweeps *p < 0.05 between white-opsin and ChR2.

To evaluate the dependence of peak current and on/off-rate on pulse-width of white light stimulation of white-opsin-HEK293 cells, we varied the shutter exposure time from 100 ms to 500 ms. Representative inward current in white-opsin and ChR2 expressing HEK293 cells upon white-light illumination with different pulse widths at fixed intensity are shown in Fig. 3a,b respectively. In Fig. 3c, we show the inward peak-photocurrent in white-opsin or ChR2 expressing cells as a function of pulse width of white-light. No statistically significant change in inward peak-current was observed with increase in pulse width in the range 100–500 ms. However, at all investigated pulse widths, the peak photocurrent for white-opsin is an order of magnitude higher than that due to ChR2. With increase in pulse width of the white-light stimulation of the white-opsin-sensitized HEK cells, the on rate decreased from ~60 ms (at 100 ms) to ~45 ms (at 200 ms) and then did not vary significantly (Fig. 3d). The quantitative comparison of on-rate of white-opsin and ChR2 in response to white light at four different pulse widths at fixed intensity (0.12 mW/mm^2^) is shown in Fig. 3d.

Next, we measured the jitter (Fig. 3e) in inward photocurrent in same cell (expressing white-opsin or ChR2) at fixed stimulation parameters. In Fig. 3f, we show the quantitative comparison of jitter time for white light induced inward current for white-opsin and ChR2 at different pulse widths. In addition to stable amplitude of photocurrent (Fig. 3c), high fidelity of latency of white-opsin in response to repetitive white light stimulation of varying pulse width was observed (Fig. 3f). Figure 3g shows the zoomed photocurrent for white-opsin and ChR2 showing different off-rates. The quantitative comparison of variation of off-rate for white-opsin and ChR2 expressing cells as a function of pulse width of white-light is shown in Fig. 3h. The off rates did not change significantly with increase in pulse width of white-light activation. However, the full-width at half maximum (FWHM) of photocurrent changed significantly with increase in pulse width of white light for both white-opsin and ChR2 (Suppl. Fig. 5). Further, FWHM of white-opsin photocurrent was found to be significantly higher than that due to ChR2. The sustained light-induced current over a longer period in case of white-opsin (as compared to ChR2) may also prove to be advantageous for generating action potential.

### Contribution of opsin components to measured white light-induced current in white-opsin

**Figure 4:**
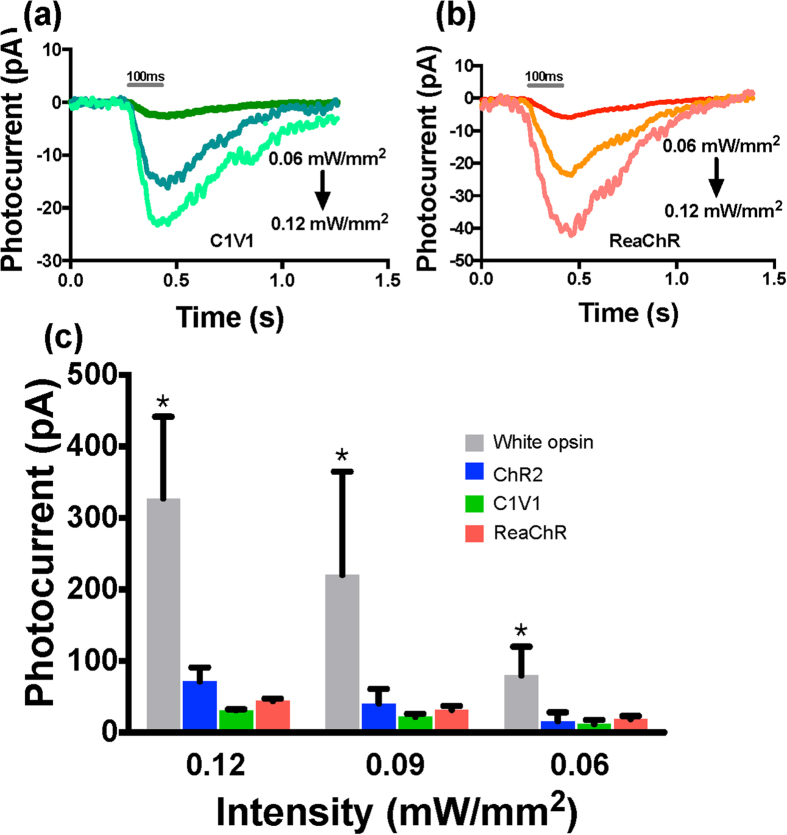
Quantitative comparison of photocurrent in cells expressing white-opsin versus ChR2, C1V1 or ReaChR in response to white-light stimulation. (**a**) Representative inward current profiles in a C1V1 expressing HEK293 cell upon white light illumination at three different intensities (0.06, 0.09, 0.12 mW/mm^2^). (**b**) Representative inward current profiles in a ReaChR expressing HEK293 cell upon white light illumination at three different intensities. (**c**) Comparison of peak photocurrent in white-opsin (N = 8 cells, 26 sweeps) vs. ChR2 (N = 8 cells, 27 sweeps), C1V1 (N = 2 cells, 12 sweeps), or ReaChR (N = 2 cells, 12 sweeps) expressing HEK293 cells at three different intensities of white-light (pulse width: 100 ms). Average ± S.D. *p < 0.01 between white-opsin and others at three different intensities.

To quantify contribution of the three opsin components (ChR2, C1V1, ReaChR) in the measured white light-induced current in white-opsin expressing cells, photocurrents in cells expressing white-opsin versus ChR2, C1V1 or ReaChR in response to white-light stimulation were measured at different intensities. Figure 2a,b respectively show representative inward current profiles measured in HEK293 cell expressing white-opsin and ChR2. In Fig. 4a,b, we show representative inward current profiles measured in HEK293 cell expressing C1V1 and ReaChR respectively. The quantitative comparison of peak photocurrent in white-opsin vs. ChR2, C1V1, or ReaChR expressing HEK293 cells at three different intensities of white-light (pulse width: 100 ms) is shown in Fig. 4c. The summation of mean values of photocurrents due to ChR2, C1V1 and ReaChR is lower than that due to white-opsin at all three measured white-light intensities. This may be attributed to better translocation of the larger fused molecule (white-opsin) across cell membrane (as compared to individual opsins) leading to higher expression that generates higher photocurrent. Furthermore, it is plausible that the fusion of the three opsins may have generated higher channel photo-conductance.

## Discussion

The motivation for using broadband activatable white-opsin is to facilitate the restoration of visual activity in ambient day-light conditions (ambient white light intensity level is ~0.01 mW/mm) without the use of any active illumination device. In order to facilitate this ambient-light based stimulation, we have developed a white-opsin that has a broad spectral excitability across the entire visible spectrum. Our results show that significant photocurrent can be reliably generated with ms on/off rate in white-opsin sensitized cells at white light intensity level close to ambient day-light condition. This should allow higher ambient white light sensitivity of white-opsin sensitized higher-order retinal neurons. The significantly reduced white light stimulation intensity required for activating white-opsin sensitized cells paves the way for ambient white light based restoration of vision in retinal degenerative diseases. Further, the ambient light based passive stimulation paradigm should provide high resolution vision restoration (not limited by pitch and number of stimulating sources) and eliminate the inconvenience of active-stimulation prosthetics. Use of white-opsin and ambient natural light will also minimize light-induced chronic damage to the opsin-expressing cells as well as residual light-sensing cells that might exist in the diseased or impaired retina.

By utilizing the whole spectrum (Suppl. Fig. 3), we expect that ambient-light can stimulate the white-opsin sensitized retinal ganglion cells (RGCs) in retina to generate action potential and activate cortical visual circuits. Suppl. Fig. 6 shows the variation of measured inward current in HEK293 cells (with high white-opsin expression) as a function of white-light intensity. Assuming maximum ChR2 expression (2 × 10^7^), Grossman *et al.*^38^ have calculated the lower bound for the generating action potential in RGC to be ~0.1 mW/mm^2^ while using blue light. White light at such intensity was found to generate ~ 80 pA in HEK293 cells expressing ChR2, and may therefore be considered as threshold photocurrent for generating action potential (also reported experimentally^39^). This threshold peak current of 80 pA (in HEK293 cells) could be achieved at white light intensity of 0.06 mW/mm^2^ in white-opsin expressing HEK293 cells (Suppl. Fig. 6). Though this threshold white light intensity (for HEK293) is still higher from the targeted ambient white light level (0.005–0.015 mW/mm^2^), ambient light is expected to generate sufficient photocurrent (for action potential) in white-opsin expressing RGCs owing to their larger size. As compared to somal surface area (~πd^2^) of 314 μm^2^ of HEK293 cells, the somal area of RGCs is known^40^ to vary from 1105 μm^2^ (Type I) to 4047 μm^2^ (Type II) which is larger by a factor of ~3.5 to 13. Considering the lowest limit, the photocurrent graph (black) for white-opsin expressing HEK293 cell is normalized by multiplying the increased-area factor (3.5) to obtain the expected photocurrent (dashed red line) in white-opsin sensitized RGCs. The dashed vertical black line shows the ambient light level (red rectangle, Suppl. Fig. 6). Inclusion of dendritic area of RGC will further lower the threshold ambient light intensity required to generate action potential in white-opsin expressing RGC and white light illumination. In case greater surface area of RGC does not significantly enhance photocurrent at ambient white light level, the ambient light can be concentrated so as to increase the light intensity reaching the retina. However, the exposure geometry and spectral environment will determine the photobiological effects on the human eye^41^. Therefore, to avoid damage to residual photoreceptors and retinal pigment epithelium of partially-blind patients, the surrounding light intensity (and exposure) has to be limited within the maximal permissible exposure^42^.

## Conclusions

To conclude, our results clearly demonstrate that an order of magnitude higher photocurrent is generated in the white-opsin sensitized cells as compared to that in ChR2, C1V1 or ReaChR expressing cell when activated using same white-light intensity. We believe that expression of white-opsin in higher order retinal cells of photodegenerated retina will lead to resensitization of the retina towards ambient white light and enable high-resolution vision restoration. However, the dynamic range and photosensitivity of opsins needs to be further improved to realize vision restoration by low levels of ambient light. The success of the ambient white light based optogenetic stimulation of retina will pave the way for minimally-invasive, high resolution and device-free treatment of photodegenerative diseases. This broadband white-opsin and white-light stimulation approach will also open new areas for application of optogenetic stimulation by ambient light.

## Methods

### White-opsin synthesis

For construction of white-opsin, a fusion of multiple opsin-encoding genes (ChR2, C1V1, ReaChR) was carried out using MultiSite Gateway® Technology^43^ (Life Technologies). To generate four entry clones, four PCR products (promoter, ChR2, C1V1, ReaChR) flanked by specific attB sites and three donor vectors were used in separate BP recombination reactions. LR recombination of the four entry clones and the destination pDEST™ R4-R3 Vector II was used to create the white-opsin construct.

### Cell culture

HEK293 cells were transfected with white-opsin or ChR2 constructs using lipofectamine (Life Technologies). After transfection, the HEK293 cells were cultured in Petri dishes and maintained in DMEM/F-12 with 10% fetal bovine serum, 0.2 mg/mL streptomycin, and 200 U/mL penicillin. The cultures were maintained at 37 °C in a 5% CO_2_ humidified atmosphere. Cells were incubated for different time periods (24 hrs, 36 hrs and 48 hrs) after transfection to allow different levels white-opsin or ChR2 expression. Visualization of the reporter (YFP) fluorescence was carried out under epifluorescence microscope.

### Optogenetic stimulation

A liquid light guide coupled to a broad-band source delivered the white light to the sample for optogenetic stimulation. Suppl. Fig. 2 shows the schematic setup for evaluating white light activation of opsin (white-opsin or ChR2) expressing cells. The white light intensity was varied by a current-controller. A power meter (PM 100D, Thorlabs) was used to quantify the white light intensity at the sample plane. The white-light pulse width was controlled by an electro-mechanical shutter, synchronized with the electrophysiology recording system (Molecular Devices). Cells, transfected with white-opsin or ChR2, were incubated with all-trans retinal (ATR, 1 μM) for 6 hours before conducting the patch clamp experiments.

### Patch-clamp recording setup

The patch-clamp recording setup consists of an Olympus upright fluorescence microscope platform using an amplifier system (Axon Multiclamp 700B, Molecular Devices). The micropipette (resistance: 3 to 5 MΩ) was filled with a solution containing (in mM) 130 K-Gluoconate, 7 KCl, 2 NaCl, 1 MgCl_2_, 0.4 EGTA, 10 HEPES, 2 ATP-Mg, 0.3 GTP-Tris and 20 sucrose. The micropipette-electrode was mounted on a motorized micromanipulator (MP225, Sutter Instruments). The extracellular solution contained (in mM): 150 NaCl, 10 Glucose, 5 KCl, 2 CaCl_2_, 1 MgCl_2_ was buffered with 10 mM HEPES (pH 7.3). Photocurrents were measured while holding cells in voltage clamp at −70 mV. The electophysiological signals from the amplifier were digitized using Digidata 1440 (Molecular devices), interfaced with patch-clamp software (pClamp, Molecular Devices). For activation of opsin (white-opsin or ChR2) expressing cells, the white-light stimulation beam was delivered by the liquid light guide. An electromechanical shutter was used in the light path for generating and controlling pulses of light. pClamp 10 software was used for data analysis.

## Additional Information

**How to cite this article**: Batabyal, S. *et al.* Broadband activation by white-opsin lowers intensity threshold for cellular stimulation. *Sci. Rep.* **5**, 17857; doi: 10.1038/srep17857 (2015).

## Supplementary Material

Supplementary Information

## References

[b1] Hartong D. T., Berson E. L. & Dryja T. P. Retinitis pigmentosa. Lancet 368, 1795–1809 (2006). 17113430 10.1016/S0140-6736(06)69740-7

[b2] Sugawara T. *et al.* Relationship between peripheral visual field loss and vision-related quality of life in patients with retinitis pigmentosa. Eye (Lond) 24, 535–539 (2010). 19590526 10.1038/eye.2009.176

[b3] Daiger S. P., Bowne S. J. & Sullivan L. S. Perspective on genes and mutations causing retinitis pigmentosa. Arch Ophthalmol 125, 151–158 (2007). 17296890 10.1001/archopht.125.2.151 PMC2580741

[b4] Mezer E. *et al.* Attitudes Regarding Predictive Testing for Retinitis Pigmentosa. Ophthalmic Genetics 28, 9–15 (2007). 17454742 10.1080/13816810701199423

[b5] Curcio C. A., Medeiros N. E. & Millican C. L. Photoreceptor loss in age-related macular degeneration. Invest Ophthalmol Vis Sci 37, 1236–1249 (1996). 8641827

[b6] Hartong D. T., Berson E. L. & Dryja T. P. Retinitis pigmentosa. Lancet 368, 1795–1809 (2006). 17113430 10.1016/S0140-6736(06)69740-7

[b7] Chader G. J. Animal models in research on retinal degenerations: past progress and future hope. Vision Res 42, 393–399 (2002). 11853755 10.1016/s0042-6989(01)00212-7

[b8] Baumgartner W. A. Etiology, pathogenesis, and experimental treatment of retinitis pigmentosa. Medical Hypotheses 54, 814–824 (2000). 10859693 10.1054/mehy.1999.0957

[b9] Hamel C. Retinitis pigmentosa. Orphanet J Rare Dis 1, 40 (2006). 17032466 10.1186/1750-1172-1-40 PMC1621055

[b10] Horsager A. *et al.* Predicting visual sensitivity in retinal prosthesis patients. Invest Ophthalmol Vis Sci 50, 1483–1491 (2009). 19098313 10.1167/iovs.08-2595 PMC2729061

[b11] de Balthasar C. *et al.* Factors affecting perceptual thresholds in epiretinal prostheses. Invest Ophthalmol Vis Sci 49, 2303–2314 (2008). 18515576 10.1167/iovs.07-0696 PMC2517253

[b12] Zrenner E. *et al.* Subretinal electronic chips allow blind patients to read letters and combine them to words. Proc Biol Sci 278, 1489–1497 (2011). 21047851 10.1098/rspb.2010.1747 PMC3081743

[b13] Chow A. Y. *et al.* Subretinal implantation of semiconductor-based photodiodes: durability of novel implant designs. J. Rehabilit Res Develop 39, 313–321 (2002). 12173752

[b14] Zrenner E. Will Retinal Implants Restore Vision? Science 295, 1022–1025 (2002). 11834821 10.1126/science.1067996

[b15] Nagel G. *et al.* Channelrhodopsin-2, a directly light-gated cation-selective membrane channel. Proc Nat Acad Sci 100, 13940–13945 (2003). 14615590 10.1073/pnas.1936192100 PMC283525

[b16] Boyden E. S., Zhang F., Bamberg E., Nagel G. & Deisseroth K. Millisecond-timescale, genetically targeted optical control of neural activity. Nat Neurosci 8, 1263–1268 (2005). 16116447 10.1038/nn1525

[b17] Miller G. Shining New Light on Neural Circuits. Science 314, 1674–1676 (2006). 17170269 10.1126/science.314.5806.1674

[b18] Zhang F., Wang L. P., Boyden E. S. & Deisseroth K. Channelrhodopsin-2 and optical control of excitable cells. Nat Meth 3, 785–792 (2006). 10.1038/nmeth936 16990810

[b19] Mohanty S. K. *et al.* In-Depth Activation of Channelrhodopsin 2-Sensitized Excitable Cells with High Spatial Resolution Using Two-Photon Excitation with a Near-Infrared Laser Microbeam. Biophys J 95, 3916–3926 (2008). 18621808 10.1529/biophysj.108.130187 PMC2553121

[b20] Zhang F., Aravanis A. M., Adamantidis A., de Lecea L. & Deisseroth K. Circuit-breakers: optical technologies for probing neural signals and systems. Nat Rev Neurosci 8, 577–581 (2007). 17643087 10.1038/nrn2192

[b21] Cao H., Gu L., Mohanty S. K. & Chiao J. C. An Integrated mu LED Optrode for Optogenetic Stimulation and Electrical Recording. IEEE Trans Bio-Med Eng 60, 225–229 (2013). 10.1109/TBME.2012.2217395 22968201

[b22] Bi A. D. *et al.* Ectopic expression of a microbial-type rhodopsin restores visual responses in mice with photoreceptor degeneration. Neuron 50, 23–33 (2006). 16600853 10.1016/j.neuron.2006.02.026 PMC1459045

[b23] Thyagarajan S. *et al.* Visual Function in Mice with Photoreceptor Degeneration and Transgenic Expression of Channelrhodopsin 2 in Ganglion Cells. J Neurosci 30, 8745–8758 (2010). 20592196 10.1523/JNEUROSCI.4417-09.2010 PMC6632886

[b24] Zhang Y., Ivanova E., Bi A. & Pan Z.-H. Ectopic Expression of Multiple Microbial Rhodopsins Restores ON and OFF Light Responses in Retinas with Photoreceptor Degeneration. J Neurosci 29, 9186–9196 (2009). 19625509 10.1523/JNEUROSCI.0184-09.2009 PMC2774241

[b25] Tomita H. *et al.* Channelrhodopsin-2 gene transduced into retinal ganglion cells restores functional vision in genetically blind rats. Exp Eye Res 90, 429–436 (2010). 20036655 10.1016/j.exer.2009.12.006

[b26] Tomita H. *et al.* Visual Properties of Transgenic Rats Harboring the Channelrhodopsin-2 Gene Regulated by the Thy-1.2 Promoter. PLoS One 4, e7679 (2009). 19893752 10.1371/journal.pone.0007679 PMC2772120

[b27] Gu L., Shivalingaiah S., Ficinski M., Wong E. & Mohanty S. Non-viral delivery and optimized optogenetic stimulation of retinal ganglion cells led to behavioral restoration of vision. *Nature Precedings* http://dx.doi.org/10.1038/npre.2012.6869.1 (2012).

[b28] Lagali P. S. *et al.* Light-activated channels targeted to ON bipolar cells restore visual function in retinal degeneration. Nat Neurosci 11, 667–675 (2008). 18432197 10.1038/nn.2117

[b29] Doroudchi M. M. *et al.* Virally delivered Channelrhodopsin-2 Safely and Effectively Restores Visual Function in Multiple Mouse Models of Blindness. Mol Ther 19, 1220–1229 (2011). 21505421 10.1038/mt.2011.69 PMC3129568

[b30] Busskamp V. *et al.* Genetic Reactivation of Cone Photoreceptors Restores Visual Responses in Retinitis Pigmentosa. Science 329, 413–417 (2010). 20576849 10.1126/science.1190897

[b31] Degenaar P. *et al.* Optobionic vision-a new genetically enhanced light on retinal prosthesis. J Neural Eng 6, 035007 (2009). 19458396 10.1088/1741-2560/6/3/035007

[b32] Guo Z. V., Hart A. C. & Ramanathan S. Optical interrogation of neural circuits in Caenorhabditis elegans. Nature Meth 6, 891–U847 (2009). 10.1038/nmeth.1397 PMC3108858 19898486

[b33] Lin J. Y., Knutsen P. M., Muller A., Kleinfeld D. & Tsien R. Y. ReaChR: a red-shifted variant of channelrhodopsin enables deep transcranial optogenetic excitation. Nature Neurosci 16, 1499–1508 (2013). 23995068 10.1038/nn.3502 PMC3793847

[b34] Kleinlogel S. *et al.* A gene-fusion strategy for stoichiometric and co-localized expression of light-gated membrane proteins. Nat Meth 8, 1083–1088 (2011). 10.1038/nmeth.1766 22056675

[b35] Hartley J. L., Temple G. F. & Brasch M. A. DNA cloning using *in vitro* site-specific recombination. Genome Res 10, 1788–1795 (2000). 11076863 10.1101/gr.143000 PMC310948

[b36] Pan Z.-H., Ganjawala T. H., Lu Q., Ivanova E. & Zhang Z. ChR2 mutants at L132 and T159 with improved operational light sensitivity for vision restoration. Plos One 9, e98924 (2014). 24901492 10.1371/journal.pone.0098924 PMC4047080

[b37] Prigge M. *et al.* Color-tuned channelrhodopsins for multiwavelength optogenetics. J Biol Chem 287, 31804–31812 (2012). 22843694 10.1074/jbc.M112.391185 PMC3442514

[b38] Grossman N. *et al.* Photostimulator for optogenetic retinal prosthesis. Paper presented at *4th International IEEE EMBS Conference on Neural Engineering: NER ‘09,* Antalya, Turkey. *IEEE*. 68–71 (2009, Apr 29-May 02).

[b39] Warren E. J., Allen C. N., Brown R. L. & Robinson D. W. Intrinsic light responses of retinal ganglion cells projecting to the circadian system. Eur J Neurosci 17, 1727–1735 (2003). 12752771 10.1046/j.1460-9568.2003.02594.x PMC2435209

[b40] Fohlmeister J. F., Cohen E. D. & Newman E. A. Mechanisms and distribution of ion channels in retinal ganglion cells: using temperature as an independent variable. J Neurophysiol 103, 1357–1374 (2010). 20053849 10.1152/jn.00123.2009 PMC2887638

[b41] Sliney D. H. Exposure geometry and spectral environment determine photobiological effects on the human eye. Photochem Photobiol 81, 483–489 (2005). 15755194 10.1562/2005-02-14-RA-439

[b42] Delori F. C., Webb R. H. & Sliney D. H. Maximum permissible exposures for ocular safety (ANSI 2000), with emphasis on ophthalmic devices. J Opt Soc Am A 24, 1250–1265 (2007). 10.1364/josaa.24.001250 17429471

[b43] Buj R., Iglesias N., Planas A. M. & Santalucía T. A plasmid toolkit for cloning chimeric cDNAs encoding customized fusion proteins into any Gateway destination expression vector. BMC Mol Biol 14, 1–17 (2013). 23957834 10.1186/1471-2199-14-18 PMC3765358

