# Supplementary material for: Broadband activation by white-opsin lowers intensity threshold for cellular stimulation
Source: Sci Rep. 2015 Dec 14;5:17857. doi: 10.1038/srep17857 (PMC4677322; doi:10.1038/srep17857)
Supplement: Supplementary Information [file srep17857-s1.pdf]

Supplementary Information

Broadband activation by white-opsin lowers intensity threshold for cellular stimulation

Subrata Batabyal[1], Gregory Cervenka[1], David Birch[2], Young-tae Kim[3], Samarendra K. Mohanty[1*]

[1]Biophysics and Physiology Lab, The University of Texas at Arlington, TX, USA.

[2]Retina Foundation of the Southwest, Dallas, TX, USA.

[3]Department of Bioengineering, The University of Texas at Arlington, TX, USA.

* To whom correspondence should be addressed.

Samarendra Mohanty

502 Yates St, 108 Science Hall

The University of Texas at Arlington,

Arlington TX 76019, USA

Fax: (817) 272 3637

E-mail: smohanty@uta.edu

Supplementary methods:

DNA Element	Flanking att Sites	PCR Primers
Promoter	attB1, attB5r	attB1 forward, attB5r reverse
ReaChR	attB5, attB4	attB5 forward, attB4 reverse
C1V1	attB4r, attB3r	attB4r forward, attB3r reverse
hChR2	attB3, attB2	attB3 forward, attB2 reverse

Supplementary Table 1. Flanking att sites for genes of interest.

The primers sequences designed to create *attB* sites for each gene of interest are listed below.

Promoter (CMV)

5' GGGG ACA AGT TTG TAC AAA AAA GCA GGC TCACCATGTT AAC ATT ATG GCC TTA GGT (Forward Primer)
5' –GGGG AC AAC TTT TGT ATA CAA AGT TGT GCT GCC CCC AGA ACT AGG GG (Reverse Primer)

ReaChR-Citrine

5'- GGGG ACA ACT TTG TAT ACA AAA GTT GCACCATGACCGGTGACCATGGTGAGCA (Forward Primer)
5' –GGGG AC AAC TTT GTA TAG AAA AGT TGG GTGCTCGAGGCTGCTCTCGTACT (Reverse Primer)

C1V1-EYFP

5' – GGGG ACA ACT TTT CTA TAC AAA GTT GCACCAT GTC GCG GAG GCC ATG GCT (Forward Primer)
5' –GGGG AC AAC TTT ATT ATA CAA AGT TGT GTC CTC CTC TTC AGC CAC CA (Reverse Primer)

hChR2-EYFP

5'- GGGG ACA ACT TTG TAT AAT AAA GTT GCACCAT GGA CTA TGG CGG CGC TTT (Forward Primer)
5'-GGGG AC CAC TTT GTA CAA GAA AGC TGG GTA TTA CTT GTA CAG CTC GTC CA (Reverse Primer)

Supplementary Table and Figure captions

Supplementary Table 1. Flanking att sites for genes of interest.

Suppl. Fig. 1. Method of white-opsin construction. (a) Schematic of normalized activation spectra of narrow-band opsin-components (ChR2, C1V1, ReaChR) of white-opsin. (b) Entry clone for each vector (ChR2, C1V1, ReaChR) to create attB sites for white-opsin expression vector. (c) Maps of opsin plasmids containing ChR2, C1V1, ReaChR, and CMV promoter sequence, ligated via LR clonase reaction. (d) Gel electrophoresis of white-opsin construct (digested by restriction enzyme Bgl II with restriction fragments).

Suppl. Fig. 2. **Schematic of experimental setup for imaging of cells expressing opsin(s) and patch clamp recordings upon activation by white light.** WL: White light lamp; L: Lens; S1 & 2: Shutters; MO: 40x Microscope objective; FL: Fluorescence excitation lamp; DM: Dichroic mirror; Ex: Excitation filter; Em: Emission filter.

Suppl. Fig. 3. **Spectrum of broadband white light.** Measured spectrum of (a) white light from lamp, used for optogenetic stimulation, and (b) outdoor ambient day light.

Suppl. Fig. 4. Correlation of opsin expression and photocurrent. Left: Three sets of bright and fluorescence images of white-opsin-YFP transfected HEK293 cells having three different expression levels and corresponding representative inward photocurrent profiles upon white-light stimulation. Scale bar: 20 µm. Right: Three sets of bright and fluorescence images of ChR2-YFP transfected HEK293 cells having three different expression levels and corresponding representative inward photocurrent profiles upon white-light stimulation. Scale bar: 20 µm.

Suppl. Fig. 5. Dependence of FWHM on white-light stimulation pulse-width of white-opsin or ChR2 expressing cells. Zoomed photocurrent for white-opsin and ChR2 showing different off-rates (and FWHM: straight lines) is shown in Fig. 3g. N= 6/opsins, 18 sweeps. Average ± S.D. *$p<0.05$ between white-opsin and ChR2.

Suppl. Fig. 6. White-light sensitivity of white-opsin. Variation of inward current as a function of white-light intensity (pulse width: 100 ms). For white opsin, N= 6 cells, 12 sweeps. Average ± S.D. For ChR2 expressing HEK cells, N= 6 cells, 15 sweeps. Average ± S.D. The dashed red line is expected inward current in RGCs (based on cell size compared to HEK) and the red rectangle shows the targeted photocurrent in the range of ambient light level (0.005-0.015 mW/mm^2).

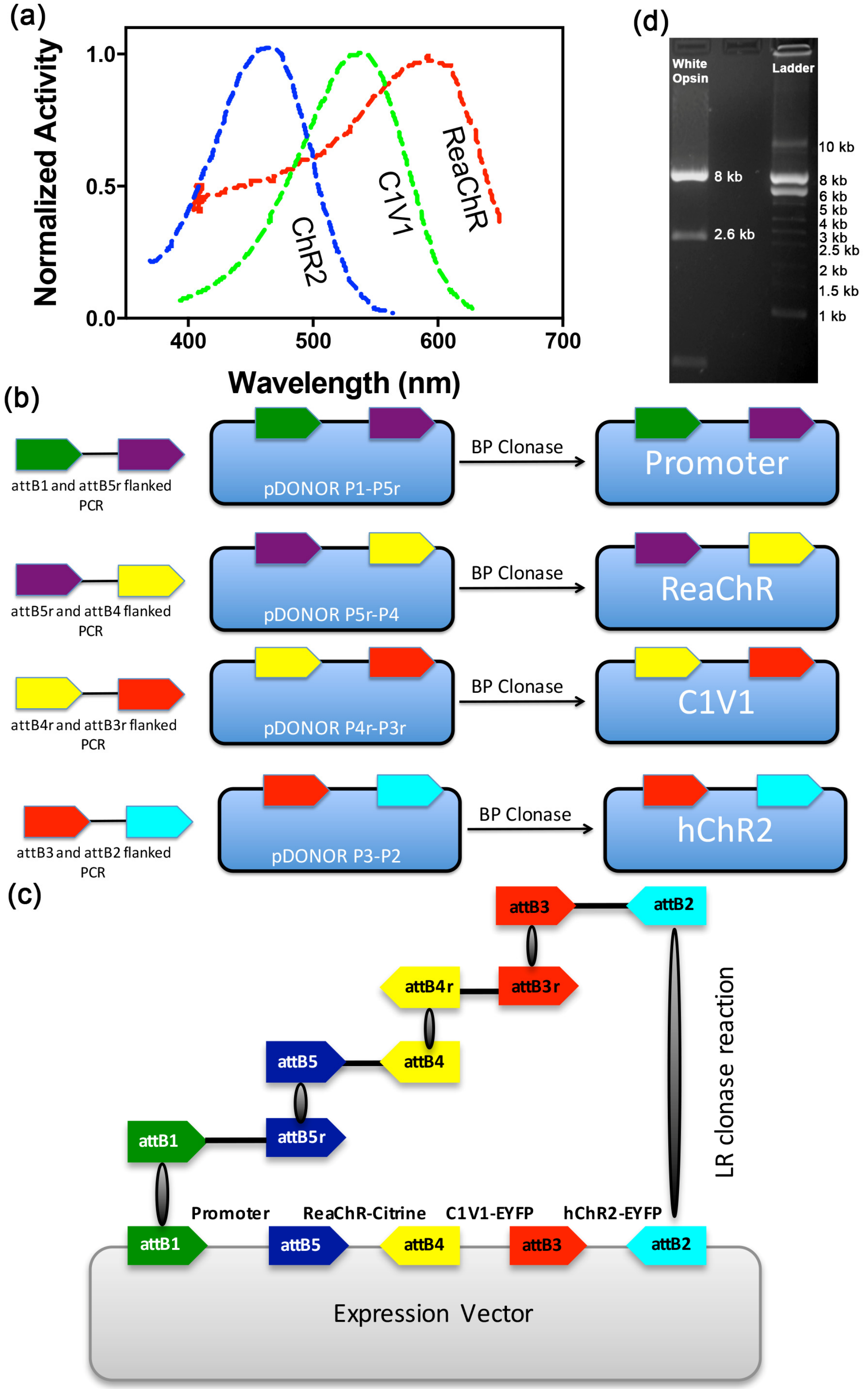

Suppl. Figure 1

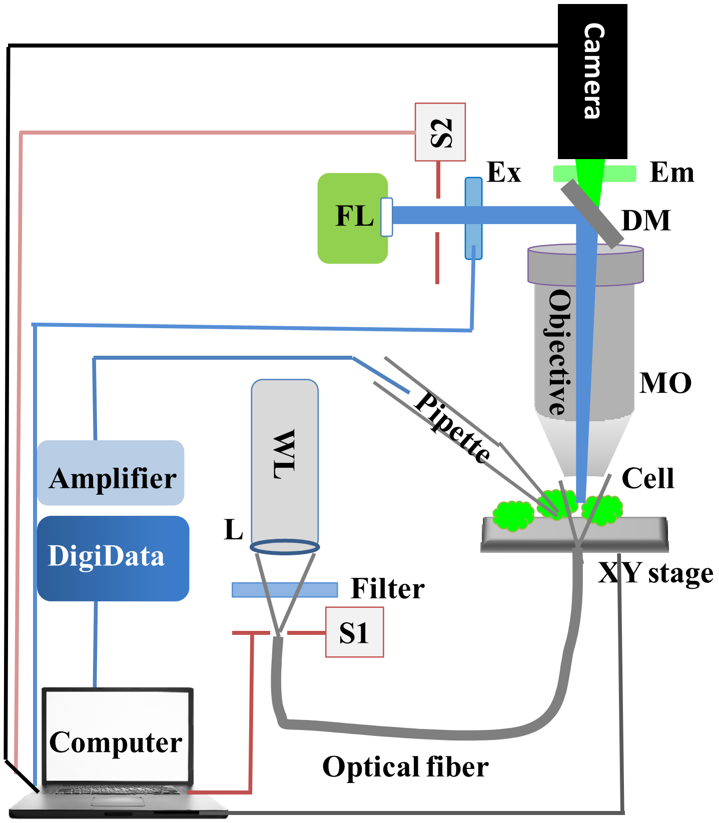

Suppl. Figure 2

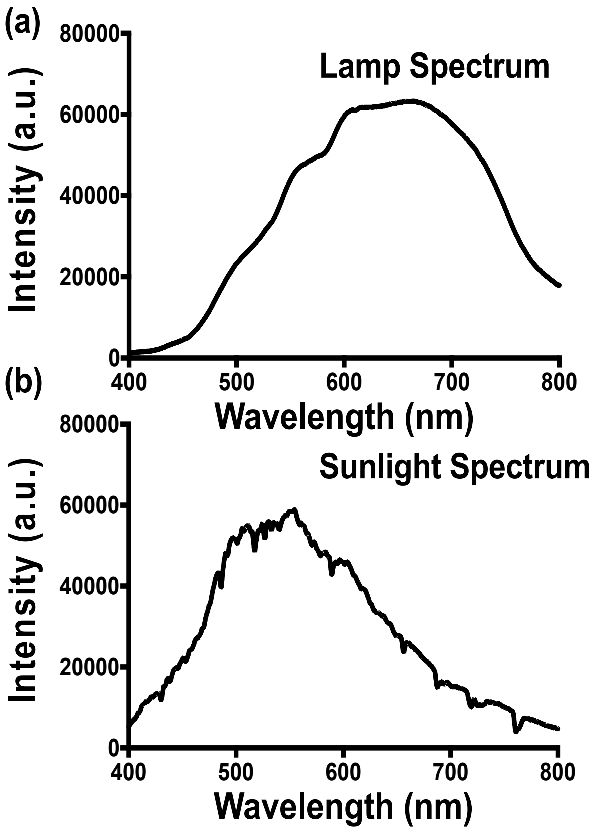

Suppl. Figure 3

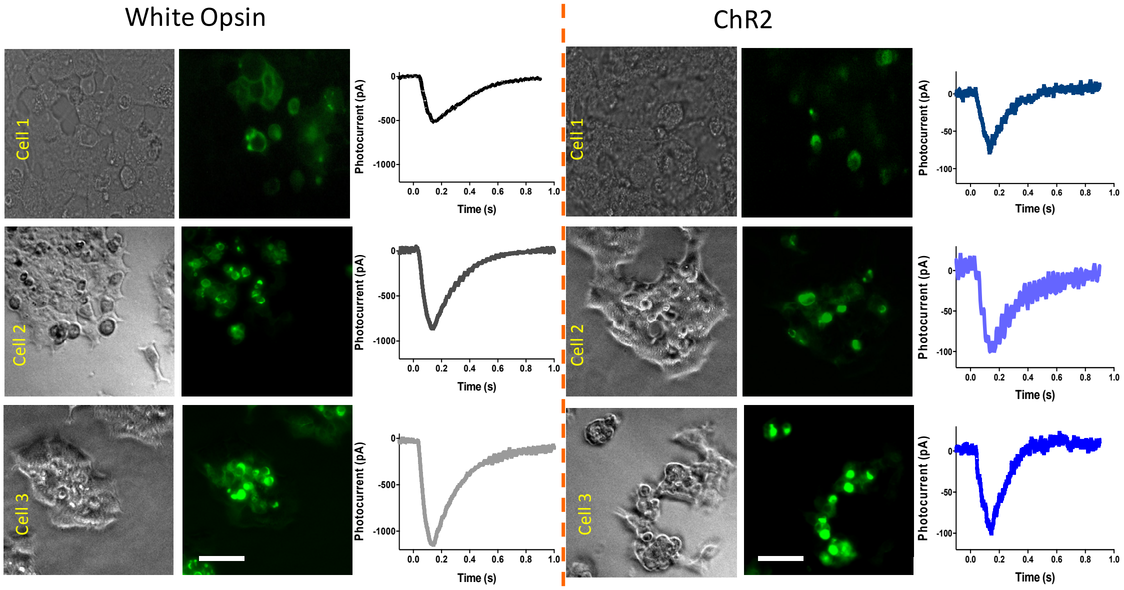

Suppl. Figure 4

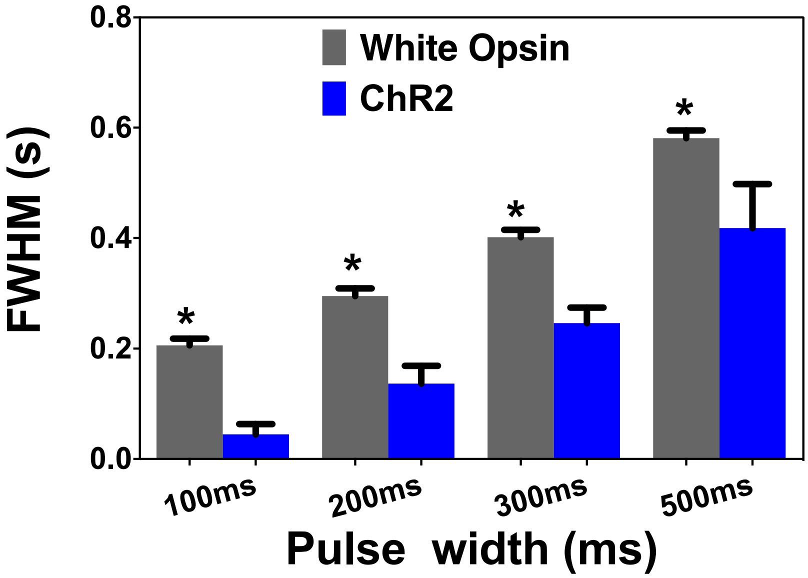

Suppl. Figure 5

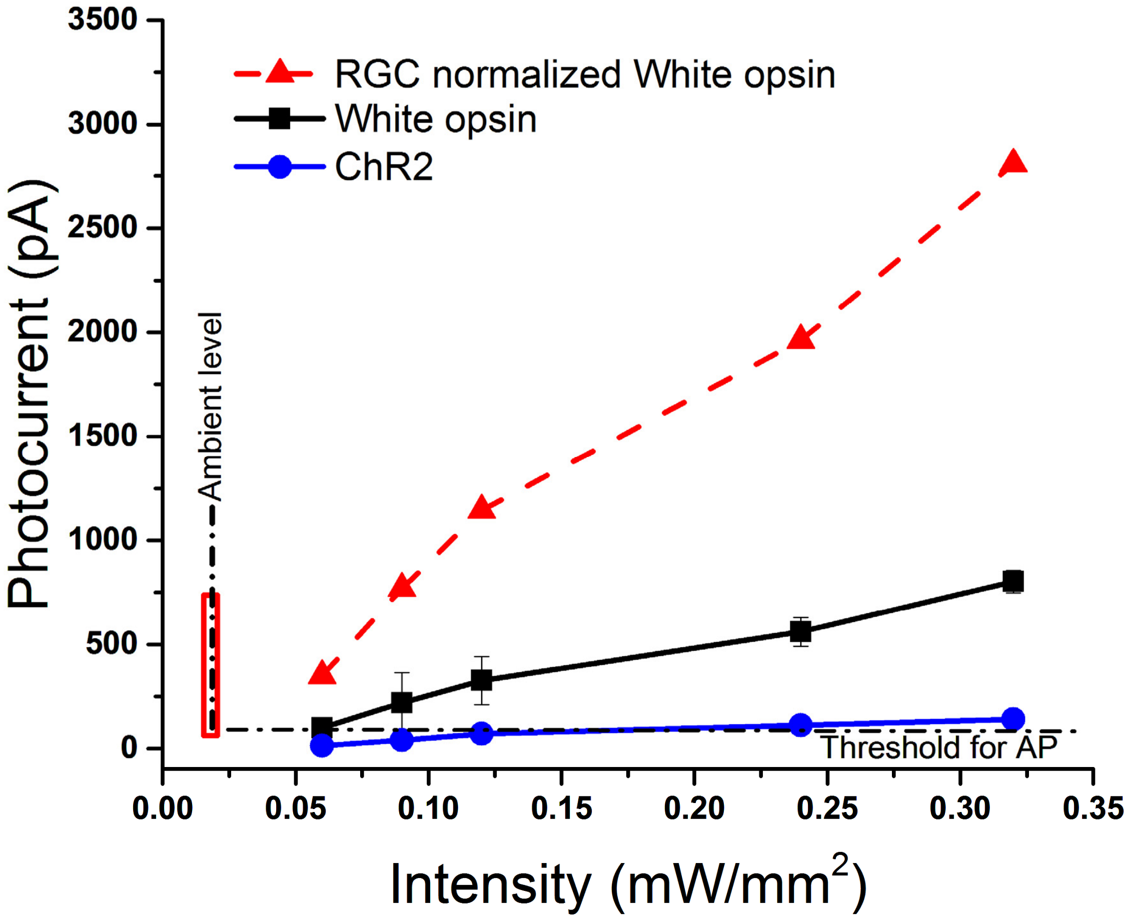

Suppl. Figure 6
